# A Rapidly Growing Evil

**Published:** 1886-02

**Authors:** 


**A Rapidly Growing Health Evil.**—It is not generally known that coal oil and gasoline stoves rapidly vitiate the air of a room for breathing purposes by the development of large quantities of carbonic dioxide. How much longer must this continue before manufacturers of such goods will obviate this new danger by inventing some form of hood and pipe for conveying this poisonous gas to the outside atmosphere? Volumes have been written concerning the ventilation of homes, and the injury that arises, especially to children and the infirm, from crowding too many people together in closed apartments, an now, with the introduction of oil and gasoline stoves in the household, a new difficulty presents itself which is not easily remedied in the endeavor to provide for health and comfort. These stoves are frequently found with several large burners in full blast, in small kitchens hardly large enough to contain air to supply the healthful requirements of one person.—*Pacific Rural.*

